# A Scalar Approach to Vaccination Ethics

**DOI:** 10.1007/s10892-023-09445-6

**Published:** 2023-09-20

**Authors:** Steven R. Kraaijeveld, Rachel Gur-Arie, Euzebiusz Jamrozik

**Affiliations:** 1 Wageningen University & Research, Hollandseweg 1, 6700 EW Wageningen, The Netherlands; 2 Edson College of Nursing and Health Innovation, Arizona State University, 550 N 3rd St., Phoenix, AZ 85004 USA; 3 Oxford-Johns Hopkins Global Infectious Disease Ethics (GLIDE) Collaborative, Oxford, United Kingdom, Baltimore, MD USA; 4 Wellcome Centre for Ethics and Humanities, Big Data Institute, University of Oxford, Li Ka Shing Centre for Health Information and Discovery, Old Road Campus, Oxford, OX3 7LF UK; 5 Royal Melbourne Hospital Department of Medicine, University of Melbourne, Parkville, 3052 Australia; 6 Monash Bioethics Centre, Monash University, Clayton, 3168 Australia

**Keywords:** Vaccination ethics, Moral reasons, Scalar morality, Public health ethics, COVID-19 vaccination, Altruism

## Abstract

Should people get vaccinated for the sake of others? What could ground—and limit—the normative claim that people ought to do so? In this paper, we propose a reasons-based consequentialist account of vaccination for the benefit of others. We outline eight harm-based and probabilistic factors that, we argue, give people moral reasons to get vaccinated. Instead of understanding other-directed vaccination in terms of binary moral duties (i.e., where people either have or do not have a moral duty to get vaccinated), we develop a scalar approach according to which people can have stronger or weaker moral reasons to get vaccinated in view of the moral good of vaccination. One advantage of our approach is that it can capture why a person might have strong moral reasons to get vaccinated with Vaccine A, but only weak moral reasons to get vaccinated with Vaccine B. We discuss theoretical strengths of our approach and provide a case study of vaccination against COVID-19 to demonstrate its practical significance.

Moral quilts made of one piece are so rare! The main thing is for the colors not to contradict each other…

—Machado de Assis (1891/1998), *Quincas Borba*

## Introduction

Some medical interventions can have health benefits for people other than the individuals who receive them. Vaccination is perhaps the clearest example of this. While some vaccines can only offer individual protection (e.g., tetanus shots), other vaccines (e.g., measles shots) can also benefit third parties, for instance by preventing or reducing the transmission of disease to others. The prospect of extra-individual health benefits associated with preventive vaccination means that one could potentially get vaccinated for the sake of others (Kraaijeveld 2020, 2023). Important moral questions emerge from this possibility. Ought people to get vaccinated to benefit others? What would constitute the grounds—and limits—of the normative claim that people should?

There have been attempts to answer these questions in light of the moral duties that people may have. Two general lines of argument for moral vaccination duties have emerged. The first line is based on the harm to others that might be caused by not getting vaccinated. Marcel Verweij (2005), for instance, has argued that individuals have a moral obligation to take precautions against infection to avoid infecting others, which may include getting vaccinated. Harm-based arguments are often applied to specific situations and appear well-suited to ground vaccination duties for people who work with, or who are otherwise closely engaged with, populations that are especially vulnerable to disease (e.g., Van Delden et al. 2008). Since vaccination is a way to prevent one from being both a victim and a vector of disease, one may have a moral obligation to get vaccinated (Jamrozik et al. 2016). According to the harm principle, originally formulated by Mill (1859/2005), the prevention of harm to third parties constitutes a necessary, if not a sufficient, condition for the restriction of individual freedoms by governments. Versions of the harm principle are therefore often invoked to justify government policies, including coercive ones, that promote vaccination so as to prevent individuals from infecting (and thus harming) others (e.g., Upshur 2002).

The second line of argument for vaccination duties is not directly based on the notion of harm or harm prevention; instead, it is grounded in non-harm-based moral principles like fairness or solidarity. Giubilini et al. (2018), for example, have argued that people have a moral duty to get vaccinated in order to contribute to herd immunity. The basic idea is that herd immunity against infectious diseases is a public good, particularly in paradigm cases where herd immunity alone can produce disease elimination. Given that people have a fairness-based duty to contribute to public goods, people therefore have a duty to contribute to herd immunity by getting vaccinated. Fairness has been proposed as a moral principle to ground vaccination duties more generally (Giubilini 2019a, b), and fairness-based approaches have been employed to justify legal requirements, including coercive measures such as vaccine mandates (e.g., Giubilini 2019a, b). Solidarity has likewise been proposed as a moral justification for vaccine mandates. It has been argued, for instance, that people ought to get vaccinated out of solidarity with others—for the sake of the wider community, but also especially for those within the community who are vulnerable to disease (Bayefsky 2018). This approach has recently been extended to vaccine mandates for the novel coronavirus disease 2019 (COVID-19), which some have attempted to justify on solidaristic grounds (e.g., Yeh 2022).^1^

Both kinds of duty-based approaches can, however, be limited in scope. To have an all-things-considered moral obligation to get vaccinated—that is, when someone may legitimately demand of you that you get vaccinated—is rarer and more circumscribed than having other-regarding reasons for getting vaccinated.^2^ There are often moral reasons to get vaccinated, but that does not necessarily entail that one has a moral duty to get vaccinated (Rieder 2021). Focusing only on moral duties may obscure part of the moral landscape. Furthermore, fairness-based duties, to the extent that they are grounded in fair contribution to public goods, are generally restricted to pathogens against which elimination via herd immunity as a public good is possible (e.g., smallpox, measles, etc.). In cases of pathogens against which elimination via herd immunity is either impossible, or where real-world transmission dynamics make it highly unlikely, such arguments may not apply or may be less persuasive.^3^ In such contexts, fairness-based arguments that appeal directly to disease elimination do not seem well-positioned to determine vaccination duties, although reducing community transmission (or rates of severe disease) through vaccination can be associated with various non-rivalrous and non-excludable goods (e.g., reduced pressure on healthcare systems) that may still be relevant to establishing moral vaccination duties (White 2021). Solidaristic approaches are vulnerable to some of the same concerns, especially when they are based on assumptions about shared common goals and shared willingness and/or ability to accept costs that may not always hold (Prainsack 2022).

Harm-based approaches to vaccination duties, on the other hand, are often complicated by the diffuse nature of the harms in question. Individuals may be ‘causally impotent’ to affect or change certain outcomes that depend on collective action (Harris and Galvin 2021). In the case of preventive vaccination, the issue of causal impotence arises when individual vaccination decisions are understood in relation to a collective goal like achieving herd immunity. An individual’s contribution to herd immunity by getting vaccinated is often ambiguous and may even be vanishingly small: group immunity is usually attained or attainable even without any given individual’s contribution (Giubilini 2019a, b). It is certainly not always clear that one will cause harm to others by not getting vaccinated. In some cases, the harm of not getting vaccinated can perhaps be determined more or less straightforwardly (e.g., for highly transmissible pathogens associated with a very high risk of severe illness). Yet this is not usually the case. If it were always true that one would cause severe harm to others by not getting vaccinated against a particular disease—or, differently put, if it were always true that by getting vaccinated, one would thereby avoid severe harm to others—a general vaccination duty grounded in harm would be more compelling, as it would better cover the moral reasons to get vaccinated. This not being the case, however, one cannot simply ground an obligation to get vaccinated *as such* in a duty to avoid causing severe harm to others. We specify severe harm, because arguing for a duty to get vaccinated to avoid any kind of harm to others can quickly become an overdemanding requirement (cf. Verweij 2005). There is a large number of potential vaccines and medical interventions that one could conceivably take to reduce risks of harm to others. In general, as Jason Brennan has pointed out, “[a]lmost everything a person does imposes some risk upon others” (2016: 4). There are many small risks of harm to others that people permissibly take every day (Hansson 2003). What we want is to move away from the distinction between the permissible and the impermissible, and to be able to determine when one has moral reasons to get vaccinated to such a degree that, all things considered, one should get vaccinated. For even in the face of causal impotence, there may still be moral reasons for individuals to act (Norcross 2020).

Therefore, rather than approach other-directed vaccination by establishing putative moral duties, we develop a scalar consequentialist account that recognizes the strength of moral reasons to get vaccinated in view of the central moral *good* of vaccination, which is to prevent or reduce the chances of causing serious harm to others. In other words, when it comes to the morality of vaccination, harm is the chief moral principle at stake (Ivanković and Savić 2021). Our reasons-based account therefore focuses on the harm-based grounds for moral claims. Although principles like fairness or solidarity may also be able to ground vaccination duties in some contexts—or provide additional moral reasons—we will not discuss them in this paper.

We proceed as follows. In Sect. 2, we introduce and defend a reasons-based scalar approach to vaccination for the sake of others. The scalar approach that we propose is consequentialist and admits of degrees: depending on a number of factors, people can have stronger or weaker moral reasons to get vaccinated for the sake of others. In Sect. 3, we develop the eight factors that, we argue, co-determine the strength of reasons to get vaccinated for others, all things considered, within any epidemiological context. In Sect. 4, we provide a case study of vaccination against COVID-19 to demonstrate the practical significance of our approach. In Sect. 5, before concluding, we reflect on our account by discussing some of its strengths, implications, and potential objections.

## Scalar Morality and Vaccination

Within moral theory, there have been calls for philosophical ethics to move away from traditional moral categories of wrongness, permissibility, and obligation (McElwee 2010a).^4^ Roger Crisp and Alastair Norcross have each argued that we should abandon binary moral judgments in terms of the traditional moral categories. According to Crisp (2006), what really matters when it comes to morality is what we have *reason* to do, while Norcross (2006) has argued that we ought to view morality in terms of concepts like goodness or badness that are best understood in terms of *degrees* (e.g., it may be better or worse to Φ). Much of the discussion has centered on what the proper domain of moral philosophy—and the primary concern of philosophical ethics—ought to be (McElwee 2010a). As far as we know, insights from these debates have not previously been extended to vaccination ethics, even though the complexity of real-world epidemiology appears, at least intuitively, to lend itself to an analysis based on (multiple) moral reasons rather than (binary) duties. In this paper, we therefore provide a rigorous evaluation of how a reasons-based account can enlighten debates in vaccination ethics.

Building on Michael Slote’s (1985) notion of scalar morality, Norcross argues that “actions should be evaluated purely in terms that admit of degrees” from the point of view of a consequentialist (Norcross 2006: 217). While goodness and badness are clearly a matter of degree, Norcross argues that rightness and wrongness should also be evaluated in terms that admit of degrees. Instead of giving an account of right action whereby an action is *demanded* by morality (e.g., because one has a moral obligation), consequentialists ought to give an account of which states of affairs are good and “which actions are better than which other possible alternatives and by how much,” so that the fundamental moral fact is “how good [an action] is relative to other available alternatives” (Norcross 2006: 228). The idea is that, once a range of alternative actions has been evaluated in terms of goodness, this exhausts the morally relevant facts about the alternatives. The appeal of this approach is that it avoids the problematic metaphysical and epistemic issue of needing to determine the precise threshold or cutoff point that would distinguish between either having or not having a moral duty to get vaccinated.

It must be noted that reasons-based consequentialist approaches have been criticized on the grounds that they cannot replace traditional moral categories of wrongness, permissibility, and obligation (McElwee 2010a). Gerard Lang (2013), for example, has argued that utilitarians cannot easily forego deontic assessment. It may be argued that we should wish to retain the notion of moral obligation, as it aligns closely with ordinary moral judgments and our everyday moral talk. However, Brian McElwee (2010b) has offered a compelling way to address this criticism, so that reasons-based approaches may still accommodate traditional moral categories. Adopting his argument here, if we find that there are pervasive moral intuitions and moral judgments—that is, strong social expectations—about obligations to get vaccinated, then we may simply recognize this state of affairs as providing a(nother) moral reason for vaccination. At the same time, one must be careful not to accept social expectations or widespread moralization as inherently justified; upon careful reflection, even an issue that is commonly perceived as morally charged may be properly understood as being morally neutral (Kraaijeveld 2022; Kraaijeveld and Jamrozik 2022). Much of the criticism of reasons-based consequentialist approaches has questioned their capacity to systematically ground morality or to displace all traditional moral categories (cf. McElwee 2010b). We do not wish to argue along these lines, however. We are interested in showing more specifically that the application of a reasons-based consequentialist approach can provide a compelling way to address the question of whether and to what extent people should get vaccinated for the sake of others.

To this end, we combine Crisp’s notion of what we have (moral) reason to do, with Norcross’s idea of the scalar goodness of actions. That is, a person’s act of getting vaccinated for the sake of others (or not) may be better (or worse). We take it that the moral good of vaccination is to prevent or reduce serious disease-related harm to others.^5^ Importantly, what makes the act of getting vaccinated (or not) morally better (or worse) is constituted by (1) an agent’s responsiveness to the (moral) reasons that she has, (2) the actual outcome in view of the good of vaccination, and (3) the available alternative actions that might achieve that good in the same or a similar way. For instance, if an available vaccine cannot prevent harm to others (e.g., because it has no effect on reducing transmission), then the moral good of vaccination cannot be attained by getting vaccinated. This is why both moral reasons and the specific affordances of specific kinds of vaccines, as well as wider epidemiological circumstances, are important to take into consideration. We generally maintain that it is better to get vaccinated for the sake of others the more one has moral reason to do so, and the more the good of vaccination can only be realized through vaccination. In practice, these notions will usually, but not necessarily, be related.

More specifically, we propose the following eight probabilistic and harm-based factors as co-determining the strength of (moral) reasons to get vaccinated for the sake of others:

(A) The probability that Person *P* will at some point be infected with pathogen *Z*.
(B) The probability that *P*, if infected, will infect an individual *I* (or individuals *I*s) with *Z*.
(C) The ex-ante probability that *P*, if infected, causes severe harm to *I*/*I*s through *Z*.
(D) The degree to which *I*/*I*s can reduce the risk of contracting *Z* or the risk of severe harm caused by *Z*.
(E) The probability that *I/I*s would be infected by people other than *P*, whether or not infected by *P*.
(F) The ex-ante probability of onward chains of transmission beyond close contacts of *P*.
(G) The probability of recovery through treatment for disease(s) caused by *Z*.
(H) The sum of costs (e.g., material, risks) for *P* to vaccinate against *Z*.

We will further develop factors A–H in the following section. While each factor is important, none is sufficient on its own for an all-things-considered answer to the question of whether it is better to get vaccinated within a given context.^6^ Rather, we argue that the different factors, considered together, co-determine the strength of moral reasons to get vaccinated based on the details about the specific pathogen in question, the severity of the associated disease, the transmission dynamics, the available vaccines, the relevant non-pharmaceutical interventions (NPIs), and other contextual factors as specified by factors A–H. Practically, the ethical discussions based on our approach will center on the moral reasons to get vaccinated against a specific disease with a particular vaccine, given that this is the tangible action at stake. It may therefore be that there are strong moral reasons for a person to get vaccinated against Disease X with Vaccine A, but that there are only weak moral reasons to get vaccinated against Disease Y with Vaccine B. There may even be more reason to get one rather than another available vaccine for the same disease (e.g., if it has a larger potential effect on reducing chances of transmission, has a better safety profile, etc.).

Our approach allows us to analyze most epidemiological contexts and to draw conclusions about the strength of pertinent moral reasons for vaccination. The greater the strength of the moral reasons to get vaccinated, the more one ought to do so. With this in mind, the contents of factors A–H are not presumed or even expected to be static, nor will they always apply in the same ways to all people. Moral reasons for getting vaccinated may differ between individuals and may change over time, depending on the ways in which the eight factors are instantiated.

## Factors that Co-determine Moral Reasons to Get Vaccinated

In this section, we enlarge step-by-step upon the initial list of factors A–H offered in Sect. 2, which can provide stronger or weaker reasons to get vaccinated.

(A) The probability that Person *P* will at some point be infected with pathogen *Z*.

The probability that a person will be infected with a particular pathogen in the future is an important consideration. If there is zero chance that a person will become exposed to a pathogen, say, over the course of their lifetime, then this provides no reason to get vaccinated against said pathogen for the sake of others. Perhaps, then, this factor could in some cases in itself be sufficient to settle the moral question of whether one should get vaccinated: if P(A) = 0 for pathogen Z, then one does not have a reason to get vaccinated against Z. As the probability of (A) increases, however, the reason to get vaccinated becomes stronger accordingly.

Nevertheless, P(A) = 0 is unlikely to hold for many pathogens. Practically speaking, the question of vaccination for the sake of others will arise when there is at least some probability of infection (but note factor [B]). As such, the probability of (A) will be greater than 0 for most pathogens of concern. The actual probability of infection will be contingent on local epidemiology and the likely future states of local and global epidemiology, which, in turn, often depend on the degree of herd immunity against the disease in question. It should be noted that, while some authors use the term “herd immunity” to refer to a threshold at which immunity alone may be expected to produce disease elimination, the term can also refer to the indirect protection generated by immune individuals in a population, which is proportionate to the number of immune people, their average degree of immunity, and their degree of interaction with others (rather than a threshold) (Fine et al. 2011).

Herein lies the classic problem of the justification of vaccination for Person *P*, where vaccination rates are already high and local disease incidence is low or zero (and the chance of importation of a disease is low). Assuming that *P* does not travel, their probability of infection is related to the probability of importation of infections to the local population, and the probability of exposure within the population, if importation occurs—which in turn depend on factors related to the behavior of *P* (e.g., number and frequency of close contacts).

This brings us to factor (B).

(B) The probability that *P*, if infected, will infect an individual *I* (or individuals *I*s) with *Z*.

The probability that a person, when infected, will infect others will primarily depend on a person’s contact patterns (locally and/or globally). This is both a matter of sheer numbers (i.e., the number of people with whom one stands to interact), as well as a more specific matter of behavioral, social, and occupational patterns (e.g., frequent contact with people who are more likely to become infected). The extent to which one engages with vulnerable individuals (e.g., occupational contacts for healthcare workers) is particularly relevant here. When P(B) = 0, the reason to get vaccinated for the sake of others is very weak. Practically, however, the probability for most pathogens of interest will exceed zero. All else being equal, the higher the probability for factor (B), the stronger the moral reason to get vaccinated.

The effectiveness of potential vaccines is also highly relevant here. If a vaccine can prevent an infection and block transmission, then a vaccinated person will not be able to spread the infection to other people. This would mean that for that vaccine, P(B) = 0 post-vaccination, thus providing a strong *prima facie* moral reason to get vaccinated for the sake of others. Different vaccines will be associated with varying levels of effectiveness. For example, the current COVID-19 vaccines appear to do little to prevent infection and transmission—once infected, vaccinated people transmit SARS-CoV-2 at similar rates to unvaccinated people (Wilder-Smith 2021). The influenza vaccine, to provide another example, has been found to be only modestly effective. Over 14 consecutive influenza seasons from 2004 to 2005 onward, the mean effectiveness of influenza vaccines was 41%, which “stands in sharp contrast to effectiveness rates for other commonly used vaccines in clinical practice, many of which exceed 90%” (Edmond 2019). The extent to which risks of spreading infection to others can be reduced or eliminated through vaccination will therefore importantly depend on the vaccines in question.

(C) The ex-ante probability that *P*, if infected, causes severe harm to *I* or *I*s through Z.

The probability that a person, when infected, will cause severe harm to others depends, first of all, on the severity of the disease generally caused by the pathogen in question. Many infections might be mild (on average) and unlikely to result in severe harm in healthy populations (e.g., common cold viruses). For pathogens where P(C) is very low, the moral reason to get vaccinated for others is weak. Other pathogens might be associated with much more severe average outcomes (e.g., Ebola virus), giving people stronger moral reasons (next to prudential reasons) to get vaccinated. Aside from the potential severity of the disease associated with the pathogen, the contact patterns as described above for factor (B) will also play a role here. For example, increased probability of infecting others *simpliciter* will be associated with increased probability of causing severe harm to the extent that severe harm is a possible outcome for the disease in question; but more contact with those who are more vulnerable to severe harms would also increase overall risk to others.

Factor (C) probably maps most closely onto what is picked out by the harm principle or the principle of nonmaleficence when applied to vaccination ethics: the chance that one might seriously harm another human being is clearly of significant moral importance. The probability that one, when infected, causes severe harm to others furthermore appears to demand that (1) a person takes reasonable precautions to try to prevent this harm coming about, and (2) governments may—and in some cases should—intervene in individual decision-making to prevent harm to third parties. At the same time, if our account is correct, then factor (C)—as important as it is—does not cover all morally relevant considerations. Importantly, the probability of causing severe harm if infected also depends on facts about the people with whom one potentially comes into contact, as specified by the following factor (D).

(D) The degree to which *I* or *I*s can reduce the risk of contracting Z or the risk of severe harm^7^ caused by Z.

There are often precautions that people can take against infection. Individual precautions will be especially important and relevant to people who are most likely to suffer significant harm from a particular disease. The probability that vulnerable people can protect themselves against infection as such, or severe harm post-infection, is particularly relevant. If vulnerable people can effectively protect themselves against disease, then this appears to weaken *P*’s moral reason to get vaccinated to protect vulnerable people from infection. In general, as P(A) approaches 1, the moral reason to get vaccinated for the sake of others becomes weaker.

To the extent that precautions can be taken—and can reasonably be expected to be taken^8^—such precautions fall as least partly within the realm of individual responsibility. When other people refuse to (i.e., do not *want* to) take effective precautions against infection, this is not the same situation, morally speaking, as when other people cannot (i.e., are *unable* to) take effective precautions. All things being equal, one has a stronger moral reason to get vaccinated for the sake of others when the others in question cannot (rather than opt not to) protect themselves.

Aside from hygienic precautions that people might take, perhaps the most important consideration is the possibility of direct protection against a disease provided by vaccination for vulnerable people themselves. A key factor here is the effectiveness of the available vaccines (i.e., against disease) in higher-risk groups or individuals. If, for instance, available vaccines provide (1) highly effective protection for higher-risk groups, and (2) sub-optimum transmission reduction in lower-risk groups, then lower-risk groups will have less moral reason to get vaccinated for the sake of higher-risk groups. We will discuss this possibility in the case of COVID-19 in Sect. 5. If, on the other hand, vulnerable groups cannot adequately protect themselves through vaccination, but transmission by lower-risk groups can be prevented or substantially reduced, then there will be a stronger moral reason for the latter to get vaccinated for the sake of the former. This is especially compelling in the case of measles, where infants cannot receive the live measles vaccine and thus depend on (the immunity of) others for their protection (Premenko-Lanier et al. 2004), thus providing a particularly strong justification for widespread measles vaccination. This also applies to influenza, where available vaccines only moderately protect higher-risk groups (e.g., the elderly), while transmission from lower-risk groups (e.g., young people) can be reduced so as to protect higher-risk groups. This had led some to argue that influenza vaccination strategies ought to target children (Bambery et al. 2018), which generally fits with our analysis that there is a stronger *prima facie* moral reason to get vaccinated if vulnerable people cannot adequately protect themselves through vaccination.^9^

Relevant to this consideration, however, is a further one as specified by the following factor (E).

(E) The probability that *I* or *I*s would be infected by people other than *P*, whether or not infected by *P*.

A person does not operate alone in the world as a potential vector for disease. The probability that one’s close contacts would in any case be infected by someone else (i.e., by others apart from *P*) is morally relevant. This idea is closely linked to that of causal indeterminacy, which occurs “when one or more intervening agents actively interferes with or thwarts another agent’s actions” (Hale 2022: 7). If an agent’s goal is to ensure that another person does not become infected, then this goal may be more or less likely to be impeded by other agents and states of affairs. To the extent that the people whom one might infect will be infected by other people anyway, there is less reason to get vaccinated for their sake; that is, there is less moral reason to the extent that P(E) approaches 1. The clearest way to understand this is by imagining that Jackie specifically gets vaccinated to protect a relative, Uncle G. If, however, it turns out that Uncle G is going to become infected anyway (e.g., because he has many close contacts with infected people), then we might rightfully praise the other-regarding motive upon which Jackie decided to get vaccinated (see Sect. 4), but given that the reason for which she got vaccinated (i.e., to prevent Uncle G from getting infected) does not stand a chance of becoming actualized (i.e., Uncle G still gets infected no matter what Jackie does), then the moral reason for getting vaccinated to avoid Uncle G getting infected is weaker than it would be had there been at least some chance that Jackie could *actually* avoid Uncle G getting infected through her act of getting vaccinated. Jackie’s intentions are good. We can and ought to praise her for having the right intentions and acting upon them, and it may still matter morally that she is not the one who infects her uncle.^10^ However, given a situation in which her act has no effect on bringing about the desired outcome (i.e., avoiding Uncle G getting infected), it would not make sense to claim that Jackie had a strong moral reason to get vaccinated *in order to* avoid infecting Uncle G (given that this infection will inevitably come about through others). On the other hand, if one is likely to uniquely infect a person who would otherwise not be infected, then, to the extent that vaccination would prevent spreading the infection, there is a stronger moral reason to get vaccinated.

These are admittedly examples on the more extreme ends of a spectrum. Nevertheless, situations where all members of a population are likely to be infected by a virus at some point during their lifetime are not unlikely to emerge (e.g., in the case of COVID-19, as discussed in Sect. 5), so that difficult questions about the inevitability of infection (by other members of society) need to be confronted. Although we are sympathetic to the idea that what matters morally is having the right intentions (e.g., from a Kantian perspective), when the right intentions cannot bring about desired outcomes, and especially when the cost of doing so is not neutral (e.g., when there are costs to vaccination, as specified by factor [H]), then one may have good reason to seek to balance the different outcomes in order to determine what should, all things considered, be the morally preferred course of action.

Relatedly, there is the following factor (F).

(F) The ex-ante probability of onward chains of transmission beyond close contacts of *P*.

The probability that a person is involved in or catalyzes onward chains of transmission is also morally important. For instance, in scenarios where one may conceivably be part of a so-called superspreading event (Lewis 2021), there may be a stronger moral reason to get vaccinated if doing so would prevent onward transmission. Moral responsibility for distant harms is arguably less compelling than for more proximal harms, especially insofar as each person in a chain of transmission could have been involved in a different chain of transmission starting with a different first infection (i.e., insofar as conditions such as those described in the section immediately above regarding factor [G] apply). In any case, one has a stronger moral reason to get vaccinated where the probability of (F) is higher (cf. Jamrozik et al. 2016).

Another morally relevant consideration is the following, factor (G).

(G) The availability of treatment and probability of recovery for the disease caused by Z.

One aspect of ethical discussions surrounding vaccination that has not received much attention is whether and to what extent the disease(s) caused by the pathogen against which one could get vaccinated can be effectively treated.^11^ This factor is also related to discussions surrounding scarce healthcare resources and vaccination. Avoiding overburdening healthcare systems may offer an additional moral reason to get vaccinated (White 2021). As such, the extent to which serious harm may be prevented to oneself and others—not only through vaccination, but also in view of available treatments—is morally relevant. If safe and effective medicines are available, so that, even if one were to become infected, one could be effectively treated at home (e.g., with over-the-counter medicines) and avoid negative health outcomes and/or hospitalization, then this would give one less of a moral reason to get vaccinated. On the other hand, in the absence of effective treatment, one would have a stronger moral reason to get vaccinated, given that there would be no alternative way to prevent or reduce harm caused by the disease or to avoid the prospect of using scare healthcare resources.^12^

An implication of our approach, and especially for factor (G), is that moral reasons to get vaccinated may weaken (or strengthen) over time. At the beginning of the emergence of a novel pathogen, there may be no available treatment. Should a vaccine be developed before effective treatments, then one might have a stronger moral reason to get vaccinated. However, when effective treatments become available over time, this may subsequently weaken the moral reason to get vaccinated. A similar logic holds when effective treatments are available in some countries or settings but not in others.

Finally, one must consider the following factor (H).

(H) The sum of costs (e.g., material; risks of harm) for *P* to get vaccinated against Z.

The costs that are associated with vaccination are ultimately also relevant to determining moral reasons to get vaccinated. After all, costs to oneself (i.e., self-regarding reasons) must be taken into consideration alongside other-regarding reasons. The material costs for people to get vaccinated, for instance, will vary across countries and for different vaccines. Even for COVID-19 vaccines with high global priority, it is not the case that the material costs are always zero. While many countries have made vaccines free for its citizens (Glied 2021; Liu et al. 2021), not every country has done so (e.g., for India, see Bagcchi 2021). Getting vaccinated may be more costly for people in lower-middle-income countries (Makinen et al. 2012), or for people with lower incomes in high income countries. One must also take into consideration factors such as people’s ability (without negative consequences) to be absent from work to get vaccinated or to take a sick day should any side effects occur. Ideally, governments would accommodate these costs, ensuring that they are distributed fairly and that they are not so great as to prevent anyone from getting vaccinated.

More significantly, the risks of harmful side-effects associated with vaccines will be relevant to determining the all-things-considered strength of reasons a person has to get vaccinated for the sake of others. When vaccines are associated with very rare risks of only mild side-effects, this will do little to counterbalance the strength of moral reasons determined by the other factors. When the costs of vaccination—in this case the risks of side-effects—are negligible, then there is a stronger moral reason for people to get vaccinated than when such costs are higher. If the risks of side-effects are higher and associated with potentially more severe outcomes, then this ought to be taken into consideration. That is, potential costs for individuals must be balanced against the moral reasons that one has to get vaccinated (i.e., as specified by factors A–G). This is often recognized when vaccination poses a unique risk to certain individuals (e.g., to those who are allergic to some of the ingredients of a vaccine). Potential emotional and psychological costs for people who, for instance, fear needles and/or other aspects of vaccination (e.g., side effects) are also relevant here; these may sometimes provide legitimate considerations for people not to want to get vaccinated, which again have to be considered next to the relevant moral reasons.

In sum, we contend that the strength of moral reasons to get vaccinated depends on the preceding factors A–H in view of the good of vaccination.

## The Case of COVID-19 Vaccination

Let us now examine what our approach means for the question of the extent to which one ought to^13^ get vaccinated against COVID-19. In the following discussion, we rely on currently available medical and scientific data. The goal is not to develop a fixed, final account, as details are likely to change over time, to differ between populations, and so on. The purpose is rather to extend the theoretical arguments above to more practical and practicable conclusions. Some considerations may change due to further scientific and medical developments, which is to be expected. It is a strength of our approach that any potential epidemiological and medical developments pertaining to COVID-19 can in principle be accommodated by our eight general factors.

What do factors A–H stipulate in the case of COVID-19? First, the probability that one will at some point be infected with SARS-CoV-2 is high (factor [A]). The most likely outcome of the COVID-19 pandemic is that the disease will become endemic (Heriot and Jamrozik 2021). This means that, practically, almost everyone is likely to contract COVID-19 at some point, especially given that the available vaccines do not offer sterilizing immunity (Bradley et al. 2021), meaning that vaccinated people are often infected and transmit infection to others (Singanayagam et al. 2021).

Second, the probability that a person, if infected, will infect others with SARS-CoV-2 (factor [*B*]) depends on a number of things. At the beginning of the COVID-19 vaccine rollout, it appeared that the vaccines might significantly reduce transmission, which would give people a strong reason to get vaccinated for the sake of others. It has since become clear that the currently available vaccines provide only partial and short-lived protection against infection (although they provide longer-lasting benefits against the vaccinated individual’s risk of severe disease) (Swan et al. 2021; Vashishtha and Kumar 2022). While getting vaccinated might slightly reduce chances of transmission for a limited period of time, vaccinated people routinely become infected and spread the virus to others (Singanayagam et al. 2021). In fact, vaccinated individuals, once infected, have been found to transmit SARS-CoV-2 infection to others at similar rates to unvaccinated individuals (Wilder-Smith 2021). This means that getting vaccinated against COVID-19 by itself does not significantly decrease the probability of infecting others. This state of affairs deflates the strength of other-directed reasons for getting vaccinated against COVID-19, to the extent that doing so would have little impact on factor (*B*). Regardless of what we now know about the COVID-19 vaccines, some people may have acted, or are still acting, on the mistaken belief that by getting vaccinated, they cannot subsequently infect others. Aside from the potential deleterious consequences of this mistaken belief (e.g., by relaxing vigilance. one might increase the chances of infecting others), this raises important questions about public health communication. People ought to be properly informed by health officials so that they can base their actions on reasons that track the effectiveness of vaccines and their effects on reducing transmission.

Beyond vaccination, there are precautions and mitigation strategies that individuals can take to significantly reduce chances of transmission to others. If rapid tests are readily available, then being quick to get tested and in any case to effectively isolate in the case of symptoms can prevent one from infecting other people. Contextual factors pertaining to testing and isolating are important and will differ between people, such as the number of people with whom one shares a living space, whether one can isolate without fear of potential negative consequences (e.g., for employment), and so on. In general, given the relatively small role of asymptomatic spread for COVID-19 (Byambasuren et al. 2021), and assuming that one is quickly tested at the first appearance of symptoms and/or can effectively self-quarantine in response to any symptoms, it appears that the risk of an individual infecting others with SARS-CoV-2 is, or can be made, relatively small in this way. The risk can furthermore be mitigated by an individual by taking these precautions prior to engaging in activities during which one might come into close contact with (1) large groups of people, and/or (2) particularly vulnerable people.

The ex-ante probability of onward chains of transmission beyond close contacts (*F*) is also relevant here. One way in which this idea has been formulated is in terms of R—the reproduction number (or ratio) of the virus. R represents the average number of cases that are expected to occur as a result of infection by a single individual (Aronson et al. 2020). The reproduction number will thus be an important—but rough—indicator of the probability of onward chains of transmission from an infected individual. The reproduction number is a rough indicator in the sense that, while R may be greater than 1 for a population (e.g., within a country), this does not necessarily mean that this number will apply to every individual. As previously discussed, there are mitigation strategies that individuals can—and arguably should—take as soon as they experience any signs of illness. When individuals respond adequately (e.g., by self-quarantining), then they may still limit their onward chain of transmission.

Third, the ex-ante probability that one, if infected, causes severe harm to others by infecting them with SARS-CoV-2 (factor [*C*]) will likewise depend on a number of factors, which are also related to other *prima facie* reasons, including the preceding point about onward transmission. Assuming that not all individuals whom one might infect will suffer severe disease (that is, that the total sum of infections is higher than the sum of infections leading to serious illness), then by taking precautions to significantly reduce the probability of transmission *as such* and as previously described, one can thereby also reduce the probability of causing severe illness by spreading SARS-CoV-2. Those considerations pertain to the person who becomes infected. What about those who might be infected in turn? Can they do anything to mitigate the risk of severe harm?

The extent to which would-be infected others can reduce the risk of contracting SARS-CoV-2 or the risk of severe harm caused by it (factor [*D*]) is affected both by the degree of risk that the individuals in question generally face from COVID-19, as well as the degree to which vaccination reduces their chances of severe illness. In the case of COVID-19, it appears that most people at risk can get vaccinated against COVID-19 and thereby reduce their chances of serious illness. To some extent, as related, one can also reduce the risk of infecting at-risk others by taking individual precautions (e.g., in terms of hygiene, distancing, isolating). If one has significant amounts of contact among vulnerable people, the preceding might be more difficult to attain (also depending on the nature of the interactions). By getting vaccinated, one might further reduce risk of transmission to some degree, although this indirect benefit to others quickly wanes over time (Swan et al. 2021). In any case, the fact that people at risk can protect themselves against serious illness from COVID-19 arguably weakens the reason others have to get vaccinated based on this criterion (cf. Gur-Arie et al. 2021). At this point, getting vaccinated against COVID-19 seems to be best understood as a self-protective choice (Kraaijeveld 2020).

A further consideration here is whether there are effective treatments for those who would become seriously ill (factor [*G*]). At the time of writing, there are effective drugs for early treatment of COVID-19, including fluvoxamine (Reis et al. 2021), sotrovimab (Gupta et al. 2021), and molnupiravir (Painter et al. 2021; Willyard 2021). Should safe, effective, and affordable treatments for COVID-19 become more widely available, this would arguably weaken moral reasons to get vaccinated, given that serious harm from the disease could then be effectively mitigated.

As COVID-19 is becoming globally endemic (Feldscher 2021; Lavine et al. 2021), most people will likely be infected with SARS-CoV-2 at some point during their lifetime (and likely multiple times). Whether an individual becomes infected by Person A at Time 1 in one particular setting or by Person B at Time 2 in another setting appears to be, with the best of current knowledge, only a matter of time. The probability that an individual who is infected by Person P at a particular time would be infected anyway by a Person Z at a different time (factor [*E*]) is therefore high, thus giving people less of a reason to get vaccinated to the extent that doing so, even if it were to prevent transmission, would not entail that other people around them would avoid infection.

The sum of costs for one to get vaccinated against SARS-CoV-2 (factor [*H*]) is also relevant. Since the vaccines have been offered for free in most countries by accommodating governments, the material costs are low. The risks associated with the current COVID-19 vaccines are generally low, although there are rare known risks as well as unknown longer-term risks. Furthermore, the known risks appear to be stratified by age and gender, so that some demographics may be at relatively higher risk of adverse events, for example younger males who receive mRNA vaccines (Mevorach et al. 2021). Reasons to get vaccinated ought to be sensitive to such risks. In general, the costs of getting vaccinated against COVID-19 appear to be low, with the caveat that for some groups the risks may be higher (e.g., for young men and boys) and that there is still uncertainty regarding such risks (Kraaijeveld et al. 2022).

All in all, then, the moral reasons to get vaccinated against COVID-19 with current covid vaccines in the present context may be more complex than they appear, and different (groups of) people in different circumstances may have stronger or weaker moral reasons to get vaccinated. Overall, though, given that the effect of current vaccines on transmission is partial and short-lived, the moral reasons to get vaccinated against COVID-19—based on the harm that might be prevented to others—are not as strong as they are or would be for vaccines that are more effective at preventing infection and transmission.

## Discussion

There are several major points to consider regarding the above reflections and in relation to our scalar account. First, it is important to be able to establish when it is appropriate to hold people morally responsible for their actions (Talbert 2019), as well as when moral emotions like blame and praise—the ‘reactive attitudes’ in Peter Strawson’s (2008) terms—may be warranted. Regarding moral responsibility, two necessary and jointly sufficient conditions are generally posited for a person to be morally responsible for a given action: a *control* (or freedom) condition and an *epistemic* (or knowledge, cognitive, or mental) condition (Rudy-Hiller 2018). The control condition stipulates that a person must “possess an adequate degree of control or freedom in performing the action,” while the epistemic condition requires that a person’s “epistemic or cognitive state was such that she can properly be held accountable for the action and its consequences” (Rudy-Hiller 2018). That is, in order to be morally responsible for an action, one must both have been acting freely and also have been appropriately aware of what one was doing. For the condition of control to be met in vaccination scenarios, a person must be (or have been) free to act (or have acted) on the reasons she had to get vaccinated (or not) in order for her to be morally responsible for her actions. While the control condition appears to readily apply to vaccination—people generally appear to have control over whether or not they get vaccinated—people’s control over their actions and the underlying reasons to which such action is responsive may in some cases be undermined. When people are coerced, for instance, they may not be fully in control of their actions. When people are led by others to refuse vaccination, or when people are threatened with the loss of their jobs if they do not get vaccinated (e.g., through compulsory vaccination policies), the control condition may also not—or not fully—hold. The prospect of losing one’s job may constitute such a strong reason to get vaccinated that it makes other important reasons impotent. To the extent that people ought to be left at least some freedom to be able to exercise their moral responsibility and act upon self-chosen (moral) reasons (cf. Kraaijeveld 2020), governments need to ensure that people are not unduly coerced in their vaccination decisions either through the government’s own policies or through social pressure, for instance via news and social media (Wilson and Wiysonge 2020).

One might assume that with the immense amount of publicization and public communication surrounding the COVID-19 pandemic, people have all the requisite epistemic tools to reason about vaccination, appropriate precautions, and so on. Nevertheless, it is still important to consider the epistemic state of individuals. Some people may have been influenced by misinformation, so that their reasons and actions may be based upon inaccurate epistemic assumptions. Particularly, in cases where significant others have undue influence over someone’s beliefs about vaccination, the case may be made that the epistemic condition does not or not fully hold (e.g., when people are fully convinced of erroneous beliefs as a result of the influence of others). Not only deliberate misinformation, but also outdated medical and scientific knowledge regarding the functioning of vaccines, for instance, is relevant—especially when the public is epistemically reliant on public health communication.^14^

Acting on the ‘right’ reasons is clearly morally relevant for moral judgment. If the reason that one has to get vaccinated for the sake of others is weak, but one nevertheless gets vaccinated, then moral praise may be an appropriate response.^15^ Our approach is consistent with the idea of altruistic vaccination (Kraaijeveld 2020; Kraaijeveld and Mulder 2022); even if the reason to get vaccinated for the benefit of others is weak (e.g., given some of the factors that we have described), one may still choose to act on that reason. On the other hand, should a person get vaccinated merely to protect their own health—i.e., acting on a self-regarding reason—then that does not appear to merit moral praise.

Although we have not argued for this view, it may help to distinguish between moral reasons and moral requirements, where the latter “differ from other moral reasons in which kinds of reactions are appropriate,” so that “[s]omeone who violates a moral requirement without any adequate justification or excuse thereby does something morally wrong and becomes liable to some negative sanction (including moral condemnation, anger, or guilt),” but “[s]omeone who conforms to a moral requirement does not always deserve a reward (such as praise)” (Kingston and Sinnott-Armstrong 2018: 170). Even though our account does not hinge on this, perhaps some moral reasons for vaccination can be sufficiently strong to form moral requirements—for instance, when significant harm to others is at stake.

A worry here might be that determining the strength of moral reasons can take considerable work, especially when there are multiple and competing (weak) moral reasons at stake for a given action or decision.^16^ Having multiple weak moral reasons to act seems to offer an agent a certain amount of discretion about which course of action to take (cf. Shahar 2021). Even though determining one’s moral obligations is not always a straightforward task either (e.g., one may have competing moral obligations), the advantage of our approach—that it provides a more thorough evaluation of the strength of relevant moral reasons—may add complexity to determinations about how one ought to act. While noting this potential limitation (discussed in more detail below), in the case of vaccination, we expect that one should be able to arrive at an all-things-considered judgment concerning the strength of moral reasons to get vaccinated in most contexts, given the different factors (A–H) that we have elaborated. This seems likely especially where one would have strong moral reasons, which, in our view, is a feature of situations in which such decisions would matter most.

Second, the strength of reasons to get vaccinated for the sake of others can vary between individuals and even for an individual over time. Regarding inter-individual variation, for example, people working with vulnerable populations (i.e., healthcare workers) may have stronger moral reasons to get vaccinated than people who do not (e.g., the general population). People who travel extensively and interact with many different people may have stronger moral reasons to get vaccinated than people who remain largely confined to their homes. These are important moral considerations. It may make moral blame appropriate in some cases, where factors A–H indicate strong moral reasons to get vaccinated, yet a person fails to respond to such reasons. It may also make moral blame misguided in other cases, for instance when factors A–H offer only weak reasons to get vaccinated for others, and a person responds by not getting vaccinated. Regarding intra-individual variation over time, it may be that someone has a stronger moral reason to get vaccinated at Time T than at some later Time Z. For example, as previously discussed, if early on in a pandemic there are no available treatments against a disease but there are available vaccines that reduce transmission, then one has a stronger moral reason to get vaccinated than later on in the pandemic, *ceteris paribus*, should effective treatments have become widely available. This highlights the importance of carefully considering factors A–H and updating them with the latest information at any point in time while considering moral reasons for vaccination, as well as determining how exclusively the good of vaccination can be achieved at different times and in various circumstances.

Third, a strength of our approach is that it avoids metaphysically and epistemically vague thresholds that arise in attempts to ground moral duties. In complex epidemiological scenarios, there is no ‘magical’ cutoff point to separate one’s either having or not having a moral duty to get vaccinated. There are moral reasons to do so, as we have argued; and this seems to match intuitions and common moral talk about whether or not one should get vaccinated even when one does not have an ethical duty to do so (cf. Rieder 2021). The difference with rival views that ground vaccination duties, say, in terms of fairness is that, when fairness considerations are not sufficiently compelling to (fully) ground a vaccination duty in some Scenario A, such accounts have little else to say beyond the conclusion that one does not have a fairness-based moral duty to get vaccinated in Scenario A.^17^ This, however, seems to leave out relevant moral reasons in many cases. As such, while our moral-reasons based account may not (fully) supplant obligation-based accounts, it at least extends the moral discourse where talk about vaccination obligations ends. Importantly, there is also not necessarily a one-to-one relation between the threshold at which one has, say, a fairness-based obligation to get vaccinated, and the strength of fairness-based moral reasons to get vaccinated. While our reasons-based account can in principle accommodate such fairness-based moral reasons, it is to its strength—compared to duty-based accounts—that it can factor in and weigh, even in an all-things-considered calculus, many relevant moral reasons to get vaccinated.

Still, something more can and should be said about when, practically speaking, one should get vaccinated for the sake of others based on the moral reasons at stake. After all, this involves a binary outcome: one either does or does not get vaccinated. While the eight factors A–H co-determine the overall strength of the moral reasons one has to get vaccinated, we have argued that the prevention of harm caused to others is the good at stake when it comes to other-directed vaccination. As such, when it is very likely that one will infect others with a disease and thereby cause serious harm, one will have strong moral reasons to take appropriate measures to avoid such harm, which includes vaccination if it can significantly reduce chances of transmission—and one should respond accordingly. Only mitigating factors like the existence of safe and effective treatments (factor [G]) or having a medical contraindication and/or belonging to a group for whom risks of harm are significantly higher than for other populations (factor [H]), weakens all-things-considered reasons for getting vaccinated in this case. Still, as mentioned above, one might be concerned that a reasons-based approach complicates practical decision-making, to the extent that one must determine the (1) the reasons one has for any given action, (2) the strength of those reasons, and (3) how these reasons count toward how one ought to act in a given case (e.g., whether to get vaccinated or not). However, reasons as “consideration[s] that count in favor of or against an action” (Rieder and Bernstein 2020: 309) are a natural part of everyday (moral) decision-making. Spelling out potential moral reasons for any given action need not—at least in principle—be more demanding than elucidating the moral obligations that one might have for the same action, although we grant that there are likely to be more reasons than obligations for any potential moral action (as noted above, we consider this extension of the moral discourse to be an advantage of our account). Even so, moral obligations are not always transparent to or straightforwardly determined for an agent, nor do they always point an agent toward a singular course of action (e.g., because there may be competing obligations pulling the agent in different directions).

Finally, it should be noted that moral reasons for vaccination may not be commensurate with legal duties. There is a large literature about the relation between moral and legal obligation (e.g., Johnson 1975; Kramer 2005) that we cannot address here. Ideally, individuals would always act on the strength of moral reasons to get vaccinated. However, even when people do respond to the moral reasons that they have to get vaccinated, states may still seek to regulate vaccination (i.e., through policies and laws). For example, herd immunity against a particular disease may not be achieved or maintained even when citizens appropriately respond to the strength of moral reasons to get vaccinated. That is, the strength of moral reasons for individuals to get vaccinated may not translate to what governments ought to do at a regulatory level—for instance, to promote or safeguard public health. Regulating vaccination for the sake of reducing community transmission, for example, may in that case appeal to grounds other than (the strength of) individuals’ moral reasons. It is likely that different policymakers and/or populations will have different preferences about when such policies might be justified, especially when they are coercive in nature, but one important factor is clearly the strength of people’s moral reasons. This is because, other things being equal, restrictive or coercive policies may be more justified and justifiable when there are already strong moral reasons for people to act in keeping with such policies. In a situation where most people have only weak reasons to get vaccinated for the sake of others, based on the eight factors that we have typified in this paper, mandatory vaccination is considerably more difficult to justify from a moral perspective. Nevertheless, one might raise the criticism that it is easier to justify government enforcement of other-regarding obligations than it is to justify government enforcement of other-regarding reasons for action.^18^ While this might be true, the distinction between moral reasons and moral requirements made by Kingston and Sinnott-Armstrong (2018) again seems helpful. As previously suggested, perhaps some moral reasons (e.g., preventing a high likelihood of severe harm) are so strong that they give rise to *prima facie* moral requirements—and, rather than merely justifying negative sanctions in the form of reactive attitudes (blame, guilt, and so on), one could argue that regulatory policies and sanctions become more justifiable. Such an approach potentially avoids the over-enforcement of every other-regarding moral reason for individual action by governments, while at the same time allowing space for the regulation of some actions from a social-political—or, in the case of vaccination, from a public health—perspective. Often, but not always, actions that individuals have strong moral reasons to perform will be those for which there is the strongest moral case for regulation—and while we cannot fully address the issue here, there is scope for further work in practical ethics to determine frameworks for the appropriate incorporation of reasons-based considerations into policy deliberations.

## Conclusion

Rather than a binary, duty-based approach where people either have or do not have a duty to get vaccinated, we developed a scalar, reasons-based, consequentialist approach according to which moral reasons to get vaccinated for the sake of others may be weaker or stronger, in view of eight probabilistic and harm-based factors. We urged for sensitivity to real-world data about probabilities of infection transmission and risks of harm, as well as facts about available vaccines and the possibility of other means of risk reduction. Whether people ought to get vaccinated for others is best conceived as a matter of responding to moral reasons, with a view toward the good of vaccination—to prevent disease-related harms to others—that may be achieved more or less uniquely. We provided a case study of vaccination against COVID-19 in order to demonstrate the practical significance of our approach. This case study may have to be amended given future epidemiological and scientific developments. This, however, is a benefit of our approach. It captures the many potentially morally relevant factors that constitute the complex scenarios and developments surrounding patterns of disease, vaccines, and the harms that might be prevented, as well as the moral reasons to which they give rise.

## Footnotes

1 But see Barbara Prainsack (2022) for a compelling criticism of COVID-19 mandates grounded in the concept of solidarity.

2 We focus on moral rather than legal reasons/obligations to get vaccinated, although we will briefly discuss how these might be related in the last section of this paper.

3 It has been argued that even if one’s contribution to herd immunity is negligible, one still has a moral duty to get vaccinated (Giubilini et al. 2018). Yet this argument still presupposes herd immunity as a morally desirable goal that is achievable in principle.

4 The following discussion draws heavily on McElwee’s (2010a, b) work.

5 We specify ‘moral’ good in order to differentiate it from the individual good of vaccination, which is to prevent or reduce the chances of illness for that individual. Throughout this paper, when we refer to the ‘good’ of vaccination, it should be understood as the ‘moral’ good.

6 We focus in this paper on other-directed reasons for getting vaccinated. Clearly, there are also often self-directed or self-protective reasons to get vaccinated (cf. Kraaijeveld 2020). We will not, however, discuss self-directed reasons in this paper, so that when we refer to ‘reasons to get vaccinated’ we mean those that are other-directed.

7 We will not offer a precise definition of ‘severe’ or ‘serious’ harm here; we assume that the degree of harm is itself morally relevant. A proxy for severe harm will often be the need for hospitalization (although not necessarily so). Death and/or lifelong sequelae associated with the disease (and giving rise to suffering) are the severest forms of harm at stake. When, for example, only a short-lived and inconvenient rather than an incapacitating illness is at stake, this may still provide a reason to get vaccinated—but only if it has been balanced against every other factor, as with any other single consideration.

8 The degree to which precautions can be expected to be taken is not only relevant for individuals interacting with others (and the strength of moral reasons at stake), but it is also an important factor to be taken into consideration when it comes to state regulation of vaccination.

9 One important caveat is that we are concerned with moral reasons; to be able to respond to moral reasons, one must arguable be a (full) moral agent. It is debatable whether children, not being fully autonomous, are in such a position. In fact, we think that children cannot fully respond to moral reasons in the way that adults can, although when it comes to, e.g., teenagers, the distinctions may be more difficult to draw. This is not a major concern for our account. Vaccinating children to protect vulnerable groups may be justified on different grounds than the presence or absence of the ability of children to respond to moral reasons (Bambery et al. 2018). We are concerned here with agents who are capable of having and responding to (moral) reasons.

10 It might be especially important to leave room for and to encourage altruism and other-regarding motives [cf. a similar discussion in Kraaijeveld (2021)].

11 While this consideration may appear to overlap with factor C (the ex-ante probability that P, if infected, causes severe harm to I or Is through Z), we specifically aim to capture some of the complexity surrounding treatment options here, which may change over time and vary across different healthcare settings (e.g., between countries).

12 It must be noted, however, that states have a legal obligation “to ensure access to timely, acceptable, and affordable health care of appropriate quality” (World Health Organization 2017), and that access to healthcare itself is arguably a human right (Denier 2005). This means that there are limits to the extent to which states can place responsibility for healthcare on citizens without themselves taking responsibility (e.g., by increasing investments in healthcare).

13 Following Norcross (2006), we use the term ‘ought to’ to mean something like ‘having (moral) reason to’.

14 We think that it is clear that governments have a responsibility to ensure that the public is well informed of the current state of medical/scientific affairs, at least where this directly affects the public’s health and wellbeing.

15 It must be noted that praise (like blame) is appropriate only when a person is in fact praise*worthy* (or blame*worthy*) (Tognazzini and Coates 2021). We suggest that the sum of the strength of the moral reasons to get vaccinated, based on factors A–H, can help to determine the moral appropriateness of blame or praise, although we cannot address this matter here [but see Kraaijeveld (2023) for a brief discussion].

16 Thanks to an anonymous reviewer for raising this point.

17 That is, of course, unless one conceives of moral obligations in scalar terms. This might be done, although considering such an approach falls outside of the scope of this paper.

18 We thank an anonymous reviewer for raising this point.
